# SARS-CoV-2 lineage-specific disease symptoms and disease severity in a city in southeastern Brazil

**DOI:** 10.1590/S1678-9946202668007

**Published:** 2026-01-30

**Authors:** Flavia Cristina da Silva Sales, Carlos Augusto Prete, Leandro Abade, Lewis Fletcher Buss, Darlan da Silva Candido, Ingra Morales Claro, Filipe Romero Rebello Moreira, Erika Regina Manuli, Ligia Capuani, Camila Alves da Silva Maia, Beatriz Araujo Oliveira, Thais Coletti, Heuder Gustavo Oliveira Paião, Silvia Figueiredo Costa, Maria Cassia Mendes Correa, Fabio Eudes Leal, Kris Varun Parag, Vítor Heloiz Nascimento, Nuno Rodrigues Faria, Ester Cerdeira Sabino

**Affiliations:** 1 Universidade de São Paulo, Faculdade de Medicina, Instituto de Medicina Tropical de São Paulo, São Paulo, São Paulo, Brazil; 2 Universidade de São Paulo, Faculdade de Medicina, Departamento de Moléstias Infecciosas e Parasitárias, São Paulo, São Paulo, Brazil; 3 Universidade Estadual de Campinas, Faculdade de Engenharia Elétrica e de Computação, Departamento de Comunicações, Campinas, São Paulo, Brazil; 4 Universidade de São Paulo, Instituto de Ciências Biomédicas, Departamento de Parasitologia, São Paulo, São Paulo, Brazil; 5 Imperial College London, School of Public Health, MRC Centre for Global Infectious Disease Analysis, London, United Kingdom; 6 University of Oxford, Department of Zoology, Oxford, United Kingdom; 7 University of Kentucky, Department of Microbiology, Immunology, and Molecular Genetics, Kentucky, United States of America; 8 Universidade Federal do Rio de Janeiro, Departamento de Genética, Rio de Janeiro, Rio de Janeiro, Brazil; 9 Universidade de São Caetano do Sul, Faculdade de Medicina, São Caetano do Sul, São Paulo, Brazil; 10 Modular Research System Ltda, Departamento de Tecnologia da Informação, São Paulo, São Paulo, Brazil; 11 Instituto Nacional de Câncer, Divisão de Pesquisa Clínica, Rio de Janeiro, Rio de Janeiro, Brazil; 12 Universidade de São Paulo, Escola Politécnica, Departamento de Engenharia de Sistemas Eletrônicos, São Paulo, São Paulo, Brazil

**Keywords:** SARS-CoV-2, COVID-19, Epidemiology, Genomic surveillance, Gamma VOC

## Abstract

In 2020, Sao Caetano do Sul city, located in the metropolitan region of Sao Paulo State, Brazil, established a web-based platform to provide primary care to suspected COVID-19 patients, integrating clinical and demographic data and sample metadata. Here we describe lineage-specific spatiotemporal dynamics of infections, clinical symptoms, and disease severity during the first year of the epidemic, which included circulation of the poorly characterised Gamma variant of concern. From April 6, 2020, to April 30, 2021, we gathered clinical, demographic, spatial and epidemiological data from the city's platform. We selected and sequenced 879 PCR+ swab samples (8% of all reported cases), obtaining a spatially and temporally representative set of sequences. Daily lineage-specific prevalence was estimated with a moving-window approach, allowing inference of cumulative cases and symptom probability stratified by lineage using integrated data from the platform. Most infections were caused by B.1.1.28 (41.3%), followed by Gamma (31.7%), Zeta (9.6%), and B1.1.33 (9.0%). Gamma and Zeta correlated with larger prevalence of dyspnoea (respectively, 81.3% and 78.5%) and persistent fever (84.7% and 61.1%) compared with B.1.1.28 and B.1.1.33. Ageusia, anosmia, and coryza were respectively 18.9%, 20.3%, and 17.8% less commonly caused by Gamma, whereas altered mental status was 108.9% more common in Zeta. Case incidence was spatially heterogeneous and larger in poorer and younger districts. Our study reveals that Gamma was associated with more severe presentation of the disease, emphasising its role in the heightened mortality levels in Brazil.

## INTRODUCTION

Understanding the natural history of a new disease is crucial during an epidemic, but establishing large cohorts can be challenging in emergency situations. During the COVID-19 pandemic, some studies leveraged existing infrastructure to understand the clinical manifestation of the disease in their community^1^, while others described hospital cohorts^2-4^.

In April 2020, Sao Caetano do Sul city (SCS), located in the metropolitan region of Sao Paulo State, Brazil, implemented a digital platform to assist all suspected COVID-19 patients and to collect clinical data using standardised questionnaires. This initiative was feasible given the collaboration between the local Health Department, the University of Sao Caetano do Sul, and the University of Sao Paulo. The platform enabled systematic testing of all suspected cases and monitoring of confirmed severe acute respiratory syndrome coronavirus 2 (SARS-CoV-2) cases in the community^5^.

This allowed a detailed description of the evolution of the outbreak in the city during the first year of the pandemic in Brazil, which covers the emergence and spread of the yet poorly understood Gamma variant of concern (VOC) and its impact on the affected population's clinical manifestation. First detected in Manaus, a region that achieved a high infection rate during the first wave^6,7^, the Gamma VOC quickly spread to all Brazilian cities dramatically increasing COVID-19 deaths. Although the SARS-CoV-2 Gamma VOC wave resulted in a more deadly epidemic when compared with the first wave, little is known about the differences in clinical manifestations and disease severity of this lineage in relation to previously circulating strains, including its ancestral lineage B.1.1.28^6,8^. In this study, from a dataset containing integrated epidemiological, clinical, and genomic data, we characterise the spatio-temporal dynamics, clinical symptoms, and disease severity of the main lineages circulating in Sao Caetano do Sul during the first year of the SARS-CoV-2 pandemic.

## MATERIALS AND METHODS

### Ethics

This study was approved by the National Research Ethics Committee under protocol No. CAAE 30127020.0.0000.0068 and 32424720.8.0000.0068. The committees waived the need for informed consent and allowed the development of an unidentified analytical dataset for analysis.

### Study area description

Sao Caetano do Sul (SCS) is a municipality located in the metropolitan region of Sao Paulo, Brazil, with a population of 162,763 inhabitants^9^. It is a part of a conurbation with the cities of Sao Paulo, Santo Andre, and Sao Bernardo do Campo. The municipality has a high Human Development Index (HDI) of 0.862, with a low illiteracy rate of 1.5% compared with the 4.2% average in the Sao Paulo State.

### Corona Sao Caetano primary care programme

The Corona Sao Caetano programme initiative was implemented on April 6, 2020, designed to organise the public health response to the COVID-19 pandemic. It instructed residents to register via an online platform or telephone contact to receive a telehealth consultation for risk assessment. Participants completed an initial questionnaire online or by telephone. Those with suspected COVID-19 were subsequently interviewed by a general practitioner by telephone, who asked additional questions regarding more severe symptoms like dyspnoea and altered mental status. Pregnant individuals or patients showing alarming symptoms, such as shortness of breath, persistent fever, confusion, or lethargy, were advised to seek medical attention at the hospital. Patients without risk were instructed to stay home and perform self-collection of samples for diagnosis, receiving telehealth services for up to 14 days after the onset of symptoms^5^. Supplementary Table S1 presents the questions used to define each symptom.

### Representative selection of samples for sequencing

The Sao Caetano platform routinely collects patient addresses and identifies the corresponding neighbourhood among its 15 neighbourhoods. To obtain a comprehensive spatial representation of positive cases across the city, we randomly selected two positive samples per epidemiological week and per neighbourhood. This approach identified 1063 positive cases, covering the period from April 6, 2020, to April 30, 2021, and ensured that we captured the diversity of cases from different locations in the city.

### SARS-CoV-2 diagnosis

All samples were tested by PCR using ALTONA RealStar^®^ SARS-CoV-2 RT-PCR 1.0 Kit (Hamburg, Germany), Mico BioMed RT-qPCR Kit (Seongnam, South Korea) per manufacturer's instructions and kit availability.

### Whole genome sequencing

SARS-CoV-2 positive samples were sequenced by a tilling-amplicon multiplex PCR technique using V3 or V4 scheme as previously described^8,10,11^. Sequencing libraries were generated using the SQK-LSK109 Kit, loaded onto an R9.4.1 flow-cell on the MinION device and sequenced using MinKNOW (version 22.3.6, Oxford Nanopore Technologies, UK).

### Bioinformatic analysis

The FAST5 files generated during sequencing were basecalled, demultiplexed, and trimmed using Guppy software (version 6.0.7, Oxford Nanopore Technologies, UK). Consensus sequences were obtained by mapping FASTQ files against the reference genome of SARS-CoV-2 isolate Wuhan-Hu-1 (GenBank accession Nº MN908947) using the Minimap2 program version 2.28.0^12^ and SAMTools^13^, converting the files into BAM format. Length filtering, quality testing and consensus building were performed for each barcode using the ARTIC pipeline^14^. Genome regions with a depth of < 20-fold were not included in final consensus sequences, and are represented with N characters. Low-quality sequences, those with less than 75% genome coverage or that presented contamination were discarded from final analysis. Lineages were classified using the Pangolin COVID-19 Lineage Assigner software tool^15^ and Nextclade^16^. Consensus sequences were submitted to the GISAID platform.

### Epidemiological and clinical data

Epidemiological and clinical analyses used data extracted from the Corona Sao Caetano platform for confirmed SARS-CoV-2 cases. Non-identifiable clinical data including demographic information (age, gender, educational level, and neighbourhood) and clinical information such as symptom onset date and reported symptoms were collected via a standardised questionnaire.

In SCS, the pandemic can be divided into three phases: Phase 1: between April 4 and September 30, 2020, during which no variants of concern (VOCs) or variants of interest (VOIs) were detected; Phase 2, from October 1 to December 31, 2020, marked by the emergence of Zeta VOI (P.2); and finally, Phase 3, spanning from January to April 2021, characterised by the widespread circulation of the Gamma VOC (P.1) in Brazil.

### Inference of lineage-specific daily prevalence, effective reproduction number, and symptom probability

The daily prevalence of each lineage was estimated by applying a moving window of variable size to the daily number of sequenced samples identified as that lineage. At each instant, only samples contained in the moving window are considered for calculating the prevalence. Our algorithm uses a larger window size for periods where few samples are sequenced, avoiding excessive noise, and decreases the window size in periods with large number of sequences, increasing time precision. We chose to use a time-varying window because the crude prevalence of lineages B.1.1.28, B.1.1.33, and Zeta had multiple prevalence surges (see Supplementary Figure S1); thus, multinomial logistic regressions as used in Buss *et al*.^17^ cannot be employed to infer the continuous-time prevalence.

Inferred lineage prevalence was used to estimate lineage-specific effective reproduction number (Rt) using the EpiFilter algorithm^18^ and serial intervals reported in Brazil during the early phase of the pandemic^19^.

Integrating individual-level genomic and clinical data allowed us to infer the symptom prevalence per lineage using reported symptoms for the 879 sequenced cases. All obtained symptom probabilities were validated by reanalysing all PCR+ cases, imputing lineages based on lineage prevalences at the day of symptom onset.

A detailed description of the methods used to estimate lineage-specific daily prevalence, effective reproduction number, and symptom probabilities can be found in the Supplementary File S1.

### P-values and probability of more frequent symptoms

Hypothesis tests determined whether a given symptom was more or less frequently caused by Gamma or Zeta when compared with lineages B.1.1.28 and B.1.1.33. For more robust results, hypothesis test disregarded lineage imputation since only symptoms reported by patients with sequenced samples were considered. Infections caused by B.1.1.28 and B.1.1.33 were merged into a single class—‘B.1.1.28 or B.1.1.33’—and used Fisher's exact test with 95% confidence level to determine if the prevalence of that symptom was significantly different for Gamma or Zeta when compared with B.1.1.28 and B.1.1.33. Two hypothesis tests were performed for each symptom (one for Gamma and one for Zeta), resulting in 34 tests. Due to the large number of tests, we performed a Benjamini-Hochberg correction with a 10% false positive rate which rejected all hypothesis tests with p ≤ 0.0033.

To better interpret the p-values obtained, we also estimated the probability of a given symptom being more frequently caused by Gamma or Zeta when compared with B.1.1.28 and B.1.1.33. For that, we generated *N*samples = 10,000 samples from the posterior distribution of each symptom prevalence and calculated the empirical probability of that symptom being more frequent in Gamma or Zeta when compared with B.1.1.28 and B.1.1.33. Denoting as *p*_1_, *p*_2_,…, *p*N_S_ and *r*_1_, *r*_2_,…, *r*N_S_ respectively posterior samples of Gamma or Zeta and B.1.1.28 or B.1.1.33, the probability of that symptom being more frequent in patients infected by Gamma or Zeta is $\frac{\left|\left\{i:p_{i}\geq r_{i}\right\}\right|}{N_{samples}}$ , in which |{*i: p*i ≥ *r*i}| is the number of indices *i* such that *p*i ≥ *r*i.

### Estimation of case fatality and hospitalisation rates

Case fatality rate (CFR), case hospitalisation rate (CHR), and in-hospital fatality rate (HFR) were estimated using cases reported on the CSC platform until March 31, 2021, and severe acute respiratory infection (SARI) hospitalisations reported in SIVEP-Gripe. The latter is an open dataset that contains individual-level information such as city of hospitalisation, city of residence, date of symptom onset and outcome (recovery or death).

We selected SARI patients that lived in Sao Caetano do Sul and were hospitalised in the Sao Paulo State. We calculated the daily number of cases and deaths using the date of symptom onset, available in both datasets.

As in the procedure adopted to estimate symptom probabilities, we used a Bayesian approach to infer fatality and hospitalisation rates assuming a uniform distribution in the interval [0,1] for the probability of severe or fatal outcome *p*S. Thus, the posterior distribution for the fatality or hospitalisation rate in a given period is

*p*S|*N*S ∼ *Beta*(1 + *N*S, 1 + *N*R)


$$p_{S}|N_{S}\sim Beta\left(1+N_{S},1+N_{R}\right)$$

in which *N*S is the number of observed severe or fatal outcomes and *N*R is the observed number of recoveries. Thus, *N*S is the number of hospitalisations for CHR calculation, and the number of deaths for HFR and CFR estimates, whereas *N*R is the number of SARI recoveries for HFR inference and the difference between the number of PCR+ cases and the number of deaths for CFR and CHR calculations.

## RESULTS

### Epidemiological context

**Figure 1:**
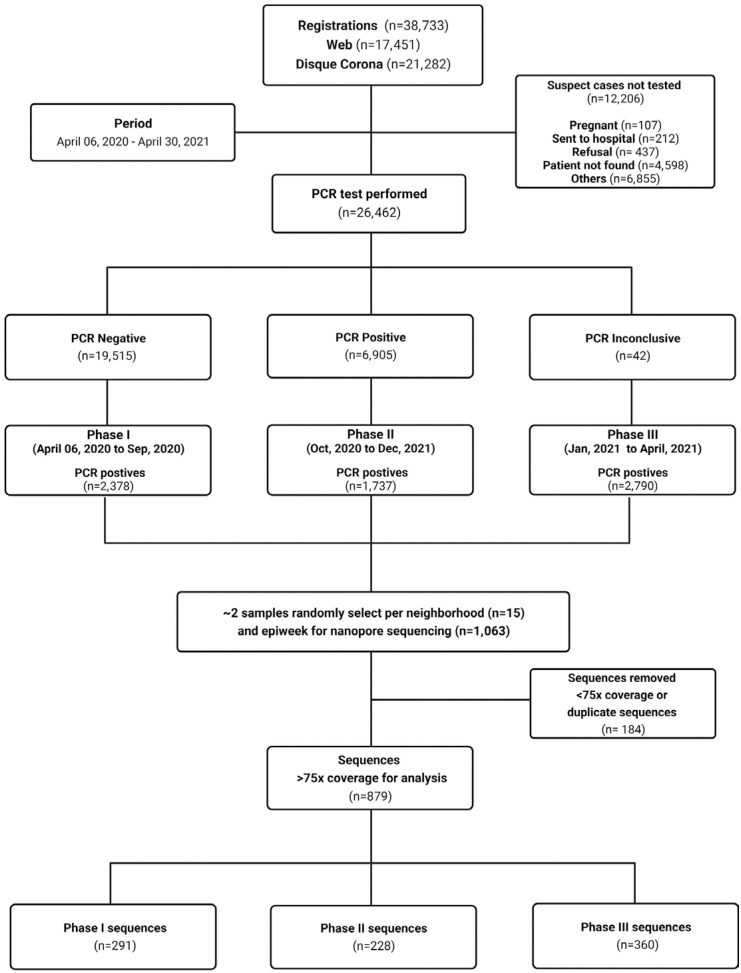
Flowchart detailing the study design. 38,733 patients registered on the CSC Platform for care. Of the 26,462 individuals who self-collected for SARS-CoV-2 diagnosis, 6,905 individuals were positive, 19,515 were negative, and 42 were inconclusive. From these positive samples, we randomly selected ∼2 positive samples per neighbourhood per epidemiological week for genome sequencing using the ARTIC protocol and the Oxford Nanopore sequencing platform. For epidemiological analyses and lineage assignment, we considered genomes with >75x coverage (n=879).

From April 6, 2020, to April 30, 2021, the SCS's Municipal Bulletin notified 10,880 COVID-19 cases. In the same period, the Corona Sao Caetano platform included 38,733 suspect cases on its digital platform. From these, 26,584 were tested for SARS-CoV-2 resulting in 6,905 positive cases that represents 63% of all notified cases in the city. Of these positive cases, we selected 1,063 samples (15.4%) for sequencing, obtaining 879 sequences with over 75x coverage (Figure 1).

Our sampling strategy for sequencing generated an epidemiologically, spatially, and temporally representative set of sequences. Table 1 compares positive and sequenced cases, showing that both groups had similar proportions of infection regardless of gender, age, and educational level. Linear correlation analysis to compare PCR+ cases by neighbourhood and epidemiological week, considering sequences with >75x coverage (Supplementary Figure S2), yielded good correlation results both spatially (Pearson's correlation coefficient =0.87) and temporally (Pearson's correlation coefficient =0.45).

**Table 1:** Demographic and clinical characteristics of patients from the Corona Sao Caetano program.

Characteristic		Total of positive cases (n=6,905)		SARS-CoV-2 genome sequences (n=879)		p-value **
		Values	Freq (%)	Values	Freq (%)	
**Sex**						0.1335
	Male	2,922	42.31%	393	44.71%	
	Female	3,983	57.68%	486	55.29%	
**Age groups**		12≤ 44≤105		12≤43≤92		0.4437
	12–19	363	5.29%	41	4.66%	
	20–39	2,618	37.94%	329	37.43%	
	40–59	2,590	37.54%	349	39.70%	
	60+	1,334	19.22%	160	18.20%	
**Educational level**						0.6166
	Illiterate	63	0.91%	10	1.14%	
	Up to primary education	1,284	18.60%	153	17.41%	
	Secondary education	3,065	44.39%	401	45.62%	
	Tertiary education	2,467	35.73%	308	35.04%	
	NA	26	0.38%	7	0.80%	
**Essential occupation**						0.7329
	Non-HCW essential job*	1,551	22.46%	204	23.21%	
	Carers	98	1.42%	13	1.48%	
	HCW	325	4.71%	46	5.23%	
	No	4,904	71.02%	609	69.28%	
	NA	27	0.39%	7	0.80 %	

NA = missing data;

* security, emergency services, supermarket, public transport, and pharmacy workers;

** Pearson's Chi-squared test.

**Figure 2:**
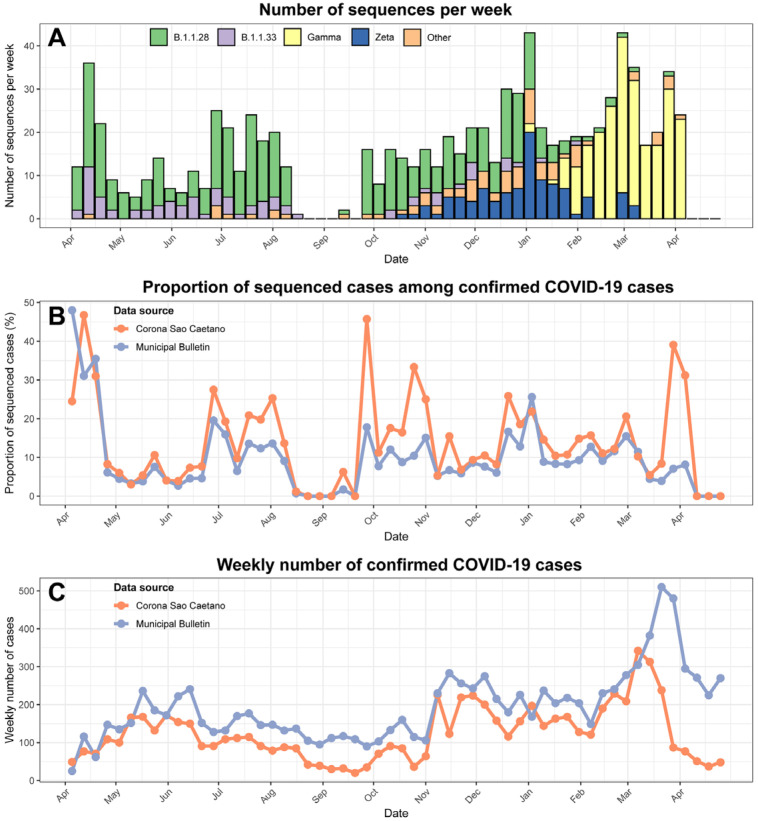
(A) Number of sequenced PCR+ samples by epidemiological week identified as the main ancestral lineages (B.1.1.28 and B.1.1.33), VOI (Zeta), and VOC (Gamma). Samples not identified as one of these lineages were assigned to the group ‘Other’; (B) Proportion of sequenced PCR+ cases among all cases reported by the municipal bulletin (in blue) or Plataforma SC (orange); (C) Weekly number of confirmed cases in SCS according to Corona Sao Caetano (by date of symptom onset) and the municipal bulletin (by date of case notification).

Figure 2 depicts the evolution of the variants over time, the proportion of sequenced cases among confirmed COVID-19 cases and the total number of confirmed cases according to the CSC Platform and the Municipal Bulletin. B.1.1.28 and B1.1.33 co-circulated in the first wave, with cases decreasing by August 2020, but slowly rebounding when Zeta VOI started to spread. The first Gamma-infected case was detected in early January, and by mid-February this VOC became the predominant variant. Of the 879 sequences obtained, B.1.1.28, B1.1.33, Zeta and Gamma were the most common lineages detected in 26.2%, 8.7%, 11.7%, and 44.1%, respectively (Supplementary Table S2). Other variants were detected in the remaining 9.3% of cases, with N.9 (23.4%), B.1.1 (18.2%), P.7 (13.0%), and the Alpha VOC (B.1.1.7) (9.1%) being the most common lineages identified.

**Figure 3:**
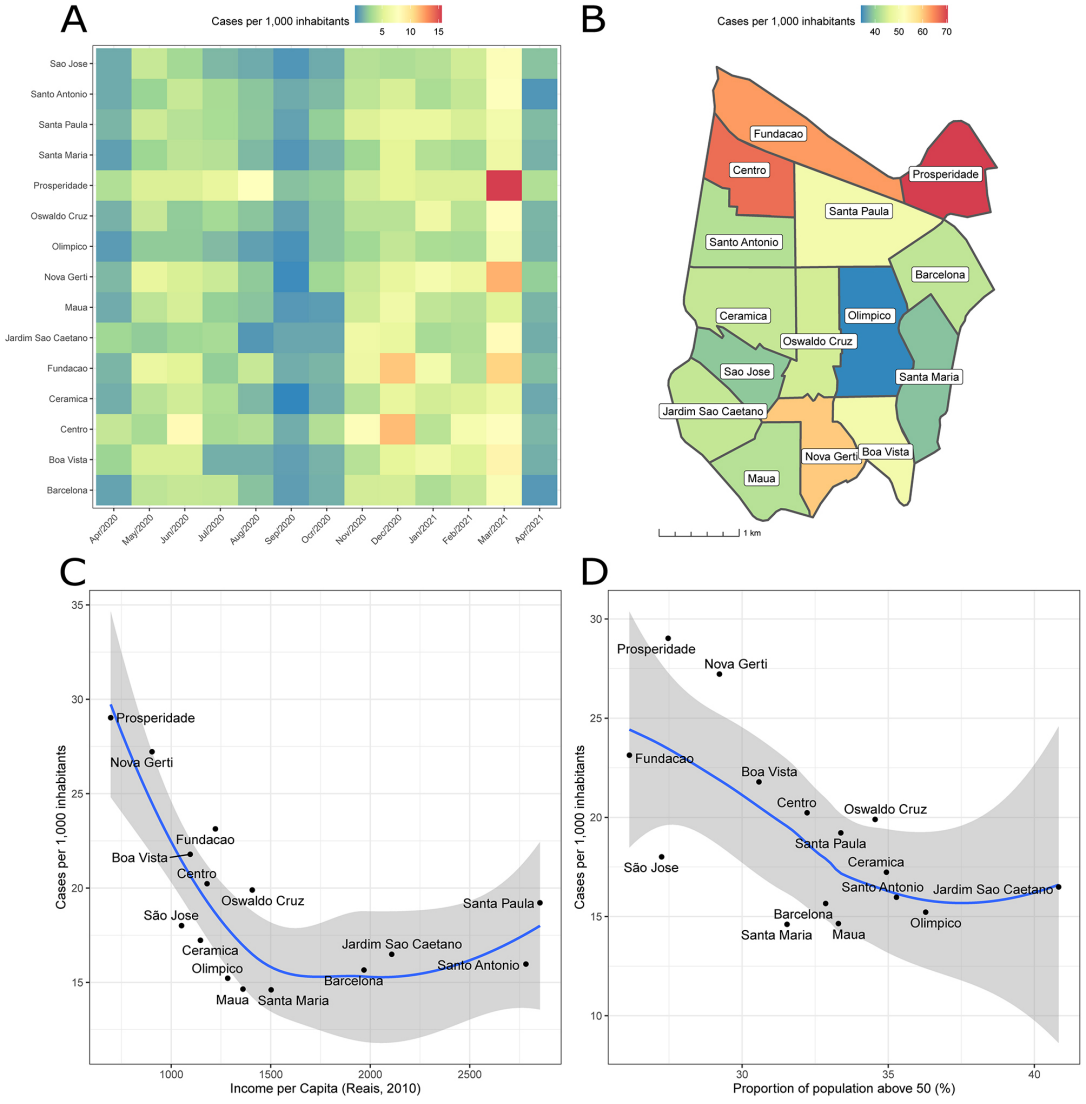
(A) Heatmap showing the monthly incidence of SARS-CoV-2 by neighbourhood in Sao Caetano do Sul from April 2020 to April 2021. Neighbourhoods are shaded according to the number of cases sampled; (B) Map of total incidence of cases (n=6,905) per 1000 inhabitants. Neighbourhoods are shaded according to the number of confirmed cases; (C and D) Total incidence in each neighbourhood by income per capita (C) and proportion of population over 50 years old (D). The curve in blue and ribbons in grey respectively represent the mean and 95% confidence intervals of a Loess regression with span=1.0.

Analysis of the monthly and total incidence of COVID-19 cases by neighbourhoods within SCS (Figures 3A and 3B) revealed that incidence varied greatly across neighbourhoods, ranging from 34 to 69 cases per 1,000 inhabitants, and this spatial heterogeneity was consistent across time. Figures 3C and 3D illustrate the total incidence in districts per average per capita income and proportion of population above 50 years old, showing that neighbourhoods with lower per capita income and younger population had higher COVID-19 case incidence.

Supplementary Figure S3 summarises the estimated cumulative incidence for each lineage . We infer that by April 30, 2021, B.1.1.28 accounted for 41.3% of the cases, followed by Gamma (31.7%), Zeta (9.6%), and B.1.1.33 (9.0%). Note that calculating the overall genomic prevalence using the estimated daily lineage-specific incidence results in a more precise measurement than simply computing the proportion of sequenced cases, as the latter underestimates prevalence of lineages circulating during periods of high incidence.

**Figure 4:**
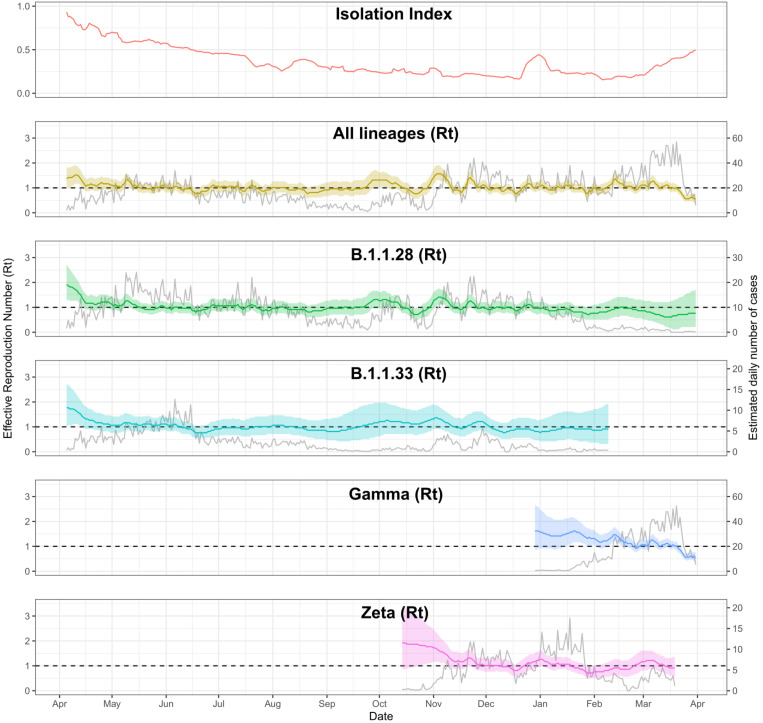
Lines show the median effective reproduction number (Rt) (y-axis on the left) estimated for each lineage, and the isolation index. Ribbons indicate 95% credible intervals. Grey curves represent the expected value of the estimated daily number of cases (y-axis on the right) by date of symptom onset caused by main lineages circulating from April 2020 to April 2021. We used the official isolation index published by the Sao Paulo State government.

Figure 4 shows the effective reproduction number for the main strains that circulated during the studied period, calculated using the inferred lineage-specific incidences. During the first and second phases of the study, Rt fluctuated between 1 and 1.5 for both major strains (B.1.1.28 and B.1.1.33). In November 2020, the B.1.1.28 lineage registered high Rt values (>1.5), coinciding with the emergence of the Zeta variant (formerly known as P.2). The Gamma VOC emerged during the circulation of the B.1.1.28 and Zeta lineage^6^. In the first two months of the Gamma epidemic, Rt oscillated between 1.0 and 1.5. When comparing Rt during the first weeks after the first detected case of each lineage, Gamma's Rt behaved similarly to other lineages despite its increased transmissibility and reinfection capability^6^, likely due to previous immunity.

Throughout the study period, overall and lineage-specific Rt oscillated around 1.0 with no large periods consistently below 1, with B.1.1.28 and B.1.1.33 showing multimodal incidence patterns. This was likely due to continuous import of cases from the neighbouring cities of Sao Paulo, Santo Andre, and Sao Bernardo do Campo, in addition to a constantly decreasing isolation index until the Gamma-dominated wave in January 2021.

### Increased disease severity caused by Gamma VOC

Descriptive analyses of case fatality rate (CFR), case hospitalisation rate (CHR), and in-hospital fatality rate (HFR) crossed data from SIVEP-GRIPE with data from the Corona Sao Caetano platform. All three disease severity indicators were higher during the second pandemic wave (phase III) compared with the previous phases (Supplementary Figure S4).

**Figure 5:**
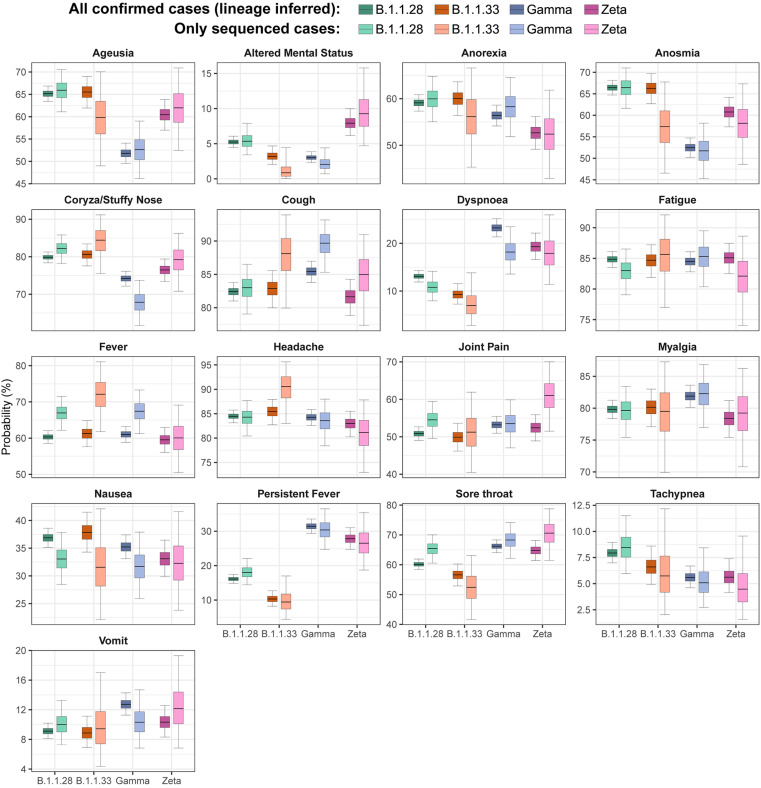
Probability of an individual infected with a given lineage reporting a specific symptom in any of the visits. Lighter boxes represent estimates obtained with only the 879 sequenced PCR+ cases; darker boxes show probabilities inferred using all PCR+ cases. Lineage for non-sequenced PCR+ cases was imputed based on the prevalence of each lineage at the date of symptom onset (see Materials and Methods for detailed description on the imputation procedure).

We performed multiple comparisons and plotted the proportion of each specific symptom according to the four most common lineages. We evaluated the 879 sequenced cases (Figure 5A), and all 6,095 cases (Figure 5B) after inputting the most probable variant according to the week of symptom onset. Both plots show that symptoms related to more severe disease, such as dyspnoea and persistent fever, were approximately two times more common among Gamma and Zeta lineages compared with the ancestral variants (B1.1.28 and B1.1.33) (see Supplementary Table S3 for exact values). Ageusia and anosmia were less common for the Gamma lineage and neurological symptoms were more common for the Zeta VOI. Due to the lack of sample size limitation, the difference in prevalence among lineages was greater when imputed lineages were used (Figure 5B).

To better understand which symptoms are more frequent, we performed one hypothesis test for each symptom and VOC/VOI, using Benjamini-Hochberg correction to adjust for the large number of tests (Supplementary Figure S5, Supplementary Table S3). The Benjamini-Hochberg correction is conservative as it assumes hypothesis tests are independent, while symptoms caused by a given lineage may be correlated. For this reason, we also highlight p-values below the 0.05 threshold in Supplementary Figure S5, and estimated the probability of a given symptom being more frequently caused by Gamma or Zeta when compared with B.1.1.28 and B.1.1.33 (Supplementary Figure S6).

To further validate our results, we also estimated symptom prevalence across age groups and study phases (Supplementary Figure S7). Disease severity as measured by dyspnoea and persistent fever was higher during the second and third pandemic phases (dominated respectively by Zeta and Gamma) for all adult age strata, whereas anosmia and ageusia were smaller during the third phase. Altered mental status (such as confusion and lethargy) was more common during the second phase for all age groups, but the difference in prevalence across phases was smaller in 0-19 and 80+ age ranges.

Notably, the null hypothesis that the prevalence of altered mental status is the same for Zeta and B.1.1.28 or B.1.1.33 was not rejected (Supplementary Table S3, Supplementary Figure S5), as the p-value was 0.08, even though Figure 5 and Supplementary Figure S7 suggest that this symptom is more prevalent for Zeta. This is because altered mental status is also more prevalent in B.1.1.28 compared with B.1.1.33, although less than in Zeta. Thus, grouping B.1.1.28 and B.1.1.33 attenuates the difference in prevalence for Zeta, increasing the p-value. For this reason, we performed six additional hypothesis tests for altered mental status comparing Zeta, B.1.1.28 and B.1.1.33 separately (Supplementary Table S4), confirming the larger prevalence of altered mental status for Zeta and B.1.1.28.

## DISCUSSION

We analysed the data obtained from a web platform created to provide care to COVID-19 cases in Sao Caetano do Sul^5^ that represents 63% of all positive cases notified in the city. In addition to epidemiological and clinical data, PCR positive samples could be retrieved for sequencing allowing a detailed description of the evolution of the pandemic in the city and the comparison of disease presentation between the different lineages. Brazil underwent one of the most significant pandemics in the world, with important regional differences. In Sao Caetano do Sul the pandemic scenario mirrored that of the metropolitan region of Sao Paulo, with a prolonged and plateau-shaped first wave followed by a more prominent second wave driven initially by Zeta lineage which was surpassed by Gamma VOC in February 2021. By December 2020, 26.6% of the population in Sao Paulo had been infected^20^.

Supplementary Figures S5, S6, S7 and Supplementary Table S3 show that disease presentation differs between lineages, as Gamma and Zeta were significantly more likely to present more severe symptoms such as dyspnoea (1.81 and 1.79-fold) and persistent fever (1.85 and 1.61-fold) compared with ancestral lineages. In an original work describing the Gamma VOC, we suggested that it caused 1.2 to 1.9 times more deaths^6^. By evaluating the infection rate in Manaus during the first and second wave using a cohort of blood donors, Prete *et al*.^20^ concluded that IHR and IFR were higher during the Gamma-dominated period.

Anosmia and ageusia were less frequent among patients infected with the Gamma VOC, as reported by Luna-Muschi *et al*.^2^ in a cohort of health care workers, whereas mental related symptoms were more present among Zeta VOI infected cases. Interestingly, Zeta infection presented more neurological symptoms, which was not previously reported. One limitation of our study is that we were unable to characterise the type or severity of neurological symptoms. Although poor mental health related to prolonged isolation may have contributed to their prevalence, the absence of an increased prevalence of neurological symptoms during the Gamma variant wave suggests that the observed increase in altered mental status may be intrinsically associated with the Zeta lineage.

Additionally, during the period when Gamma was responsible for most cases, case fatality rate (CFR), hospitalisation rate (CHR), and in-hospital fatality rate (HFR) were higher. Banho *et al*.^21^ evaluated the rate of severe and non-severe cases in Sao Jose dos Campos and detected a higher proportion of severe cases, especially among the younger population after introduction of the Gamma variant. Despite the more severe symptoms associated with Zeta infection, CHR exhibited no significant increase during the period. Although increased intrinsic disease severity may be associated with higher CFR and CHR, it does not necessarily increase HFR, as disease severity depends on the risk of hospitalisation. In analysing this using a model-based analysis of data from hospitalised patients, Brizzi *et al.* ^3^ found that in-hospital fatality rates during the Gamma-dominated wave in Brazil were primarily associated with geographic inequities and shortages in healthcare capacity rather than with the VOC.

## CONCLUSION

Our study shows that many valuable insights regarding disease transmission and symptom manifestation can only be attained by integrating epidemiological, clinical, and genomic data. Establishing an online platform to collect data associated with routine care was essential in enabling the acquisition of large, unbiased data and samples.

## Data Availability

The complete anonymized dataset supporting the findings of this study is available from https://doi.org/10.48331/SCIELODATA.I2XN3H
