# Supplementary material for: SARS-CoV-2 lineage-specific disease symptoms and disease severity in a city in southeastern Brazil
Source: Rev Inst Med Trop Sao Paulo. 2026 Jan 30;68:e7. doi: 10.1590/S1678-9946202668007 (PMC12858173; doi:10.1590/S1678-9946202668007)
Supplement: SUPPLEMENTARY MATERIAL [file 1678-9946-rimtsp-68-S1678-9946202668007-Suppl01.pdf]

REVISTA DO INSTITUTO DE MEDICINA TROPICAL DE SÃO PAULO

JOURNAL OF THE SÃO PAULO INSTITUTE OF TROPICAL MEDICINE

SUPPLEMENTARY MATERIAL

https://doi.org/10.48331/SCIELODATA.I2XN3H

SARS-CoV-2 lineage-specific disease symptoms and disease severity in a city in southeastern Brazil

Flavia Cristina da Silva Sales [1,2*], Carlos Augusto Prete Junior [3,4,5*], Leandro Abade [6], Lewis Fletcher Buss [1,2], Darlan da Silva Candido [1,5], Ingra Morales Claro [1,5,7], Filipe Romero Rebello Moreira [8], Erika Regina Manuli [1,2,9], Ligia Capuani [10], Camila Alves da Silva Maia [1,2], Beatriz Araujo Oliveira [1,2], Thais Coletti [1,2], Heuder Gustavo Oliveira Paião [1,2], Silvia Figueiredo Costa [1,2], Maria Cassia Mendes Correa [1], Fabio Eudes Leal [9,11], Kris Varun Parag [5], Vítor Heloiz Nascimento [12], Nuno Rodrigues Faria [1,5,6], Ester Cerdeira Sabino [1,2,9]

[1]Universidade de São Paulo, Faculdade de Medicina, Instituto de Medicina Tropical de São Paulo, São Paulo, São Paulo, Brazil

[2]Universidade de São Paulo, Faculdade de Medicina, Departamento de Moléstias Infecciosas e Parasitárias, São Paulo, São Paulo, Brazil

[3]Universidade Estadual de Campinas, Faculdade de Engenharia Elétrica e de Computação, Departamento de Comunicações, Campinas, São Paulo, Brazil

[4]Universidade de São Paulo, Instituto de Ciências Biomédicas, Departamento de Parasitologia, São Paulo, São Paulo, Brazil

[5]Imperial College London, School of Public Health, MRC Centre for Global Infectious Disease Analysis, London, United Kingdom

[6]University of Oxford, Department of Zoology, Oxford, United Kingdom

[7]University of Kentucky, Department of Microbiology, Immunology, and Molecular Genetics, Kentucky, United States of America

[8]Universidade Federal do Rio de Janeiro, Departamento de Genética, Rio de Janeiro, Rio de Janeiro, Brazil

[9]Universidade de São Caetano do Sul, Faculdade de Medicina, São Caetano do Sul, São Paulo, Brazil

[10]Modular Research System Ltda, Departamento de Tecnologia da Informação, São Paulo, São Paulo, Brazil

[11]Instituto Nacional de Câncer, Divisão de Pesquisa Clínica, Rio de Janeiro, Rio de Janeiro, Brazil

[12]Universidade de São Paulo, Escola Politécnica, Departamento de Engenharia de Sistemas Eletrônicos, São Paulo, São Paulo, Brazil

*These authors contributed equally to the study

Correspondence to: Ester Sabino
Universidade de São Paulo, Faculdade de Medicina, Instituto de Medicina Tropical de São Paulo, Av. Dr. Enéas Carvalho de Aguiar, 470, CEP 05403-000, São Paulo, SP Brazil
Tel: +55 11 99641 6284
E-mail: sabinoec@usp.br

Received: 18 July 2025
Accepted: 30 October 2025

Editor: Marcelo Genofre Vallada

Inference of daily lineage prevalence over time

Here we describe the procedure adopted to infer the daily lineage-specific prevalence from sparse sequencing data. Let $S[n,l]$ be the number of sequenced samples associated with lineage $l \in A$ at day n, in which $A = \{Gamma, Zeta, B.1.1.28, B.1.1.33, Other\}$. Lineages not classified as Gamma, Zeta, B.1.1.28, or B.1.1.33 are assigned into a single group 'Other'. Note that $S[n,l]$ is a sparse signal for a fixed l, being different than zero only in days where a sequenced PCR+ sample is identified as belonging to lineage l.

Define for each day $n \geq 1$ the window radius $L[n]$ as the smallest $L' \geq L_{min}$ such that

$$\sum_{m=n-L'}^{n+L'} \sum_{l \in A} S[m,l] \geq N_{min},$$

in which L_{miin} and N_{min} are predefined parameters representing, respectively, the smallest window radius and number of samples allowed. These quantities are arbitrary, and in this work we chose $L_{min} = 7$ days and N_{min} as the ceiling of the average number of samples contained in a window of radius $L_{min} = 7$ days (and length $2L_{min} + 1 = 15$ days). Hence $N_{min} = \left\lceil (2L_{min}+1)\frac{N_{total}}{\Delta t} \right\rceil = 34$, in which $N_{total} = 879$ is the total number of sequences in the period and $\Delta t = 391$ days is the total duration of the study. This method defines a window whose time-varying radius cannot be smaller than 7 days and increases until a minimum of 34 samples are included in the window.

Once $L[n]$ is computed, the prevalence of each lineage l at instant n, denoted by $\rho[n, l]$, is obtained by calculating the crude prevalence of the samples in the interval $[n–L[n], n+L[n]]$:

$$\rho[n,l] = \frac{\sum_{m=n-L[n]}^{n+L[n]} S[m,l]}{\sum_{k \in A} \sum_{m=n-L[n]}^{n+L[n]} S[m,k]}$$

Effective reproduction number estimation

We used the daily number of cases reported in the CSC Platform and the estimated daily prevalence $\rho[n, l]$ to infer lineage-specific effective reproduction

This is an open-access article distributed under the terms of the Creative Commons Attribution License.

number (R_t)) estimates. For that, we used the algorithm EpiFilter proposed by Parag *et al.*[18] with step $\eta = 0.1$. A log-normal distribution with log-mean 1.09 and log-standard deviation 0.72 was adopted as generation interval distribution based on Brazilian serial intervals measured in the early phase of the pandemic[19].

To estimate lineage-specific R_t and its confidence intervals, we calculated the daily lineage-specific incidence $I[n, l]$ as $I[n, l] = round(\rho[n, l]I[n])$, in which $I[n] = \Sigma_{l \in A} I[n, l]$ is the measured total incidence at day n (i.e., the number of PCR+ individuals that had their first symptoms at day n) and $round(x)$ is the nearest integer to x. For each $l \in A$, we apply EpiFilter (16) using $I[n, l]$ as input, obtaining a R_t estimate for lineage l. We also estimated R_t with no disaggregation by lineage by simply running EpiFilter using the daily number of cases.

In addition to R_t, $I[n, l]$ estimates also allowed us to infer the cumulative number of cases $I_C[n, l]$ caused by each lineage as $I_C[n,l] = \sum_{m=1}^{n} I[m,l]$.

Estimation of symptom probabilities

We considered that a patient with a sequenced PCR+ result infected by a given lineage had a given symptom if the symptom was reported in any of the medical visits. Credible intervals were calculated using a Bayesian approach assuming a uniform prior distribution for the probability of having the given symptom p_S in the interval [0,1]. The likelihood is $N^+|p_S \sim Binomial(N,p_S)$, in which N is the total number of sequenced PCR+ patients infected by that lineage and N^+ is the subset of this group that had the given symptom. Thus, the posterior distribution is $p_S|N^+ \sim Beta(1+N^+, 1+N\text{-}N^+)$. The quantiles of this Beta distribution are drawn to estimate median and 95% credible intervals.

To validate our results, symptom probabilities were also estimated considering all PCR+ patients, not only patients with sequenced samples. For that, we imputed the lineage that infected each patient based on the inferred lineage prevalence $\rho[n, l]$ on the day of symptom onset. Posterior distribution for p_S was obtained as described above, except that N^+ and N are estimated by $N = \sum_{i=1}^{N_{PCR^+}} \rho[n_i, l]$ and $N^+ = \sum_{i=1}^{N_{PCR^+}} \rho[n_i, l]V[i]$, in which N_{PCR} is the number of PCR+ patients, n_i is the date of symptom onset for patient i and $V[i] = 1$ if patient i had the given symptom, else $V[i] = 0$.

Supplementary Table S1 - List of questions used to determine the presence of each symptom. Some were answered by patients in the initial form (online or by telephone). Questions about more severe symptoms were asked by a physician via telephone

Symptom	Question (English)	Question (Portuguese)	Answer written by patient or physician?
Ageusia	Did you notice any change in your sense of taste?	Você sentiu alteração no paladar?	Physician
Altered mental status	Altered mental state, such as confusion or lethargy?	Alteração do estado mental, como confusão e letargia?	Physician
Anorexia	Do you have loss of appetite?	Você tem falta de apetite?	Physician
Anosmia	Did you notice any change in your sense of smell?	Você sentiu alteração no olfato?	Physician
Coryza/Stuffy nose	Do you have a blocked nose? / Do you have a runny nose?	Você está com o nariz entupido? / Você está com o nariz escorrendo?	Patient
Cough	Do you have a cough?	Você tem tosse?	Patient
Dyspnoea	Do you feel short of breath?	Você sente falta de ar?	Physician
Fatigue	Do you feel tired?	Você tem cansaço?	Patient
Fever	Have you had a fever?	Teve febre?	Patient
Headache	Do you have a headache?	Você tem dor de cabeça?	Patient
Joint Pain	Do you have joint pain?	Você tem dor nas juntas?	Patient
Myalgia	Do you have body aches?	Você tem dor no corpo?	Patient
Nausea	Do you feel nauseous?	Você tem enjôos?	Patient
Persistent fever	Persistence or increase of fever for more than 3 days, or recurrence after 48 hours without fever?	Persistência ou aumento da febre por mais de 3 dias ou retorno após 48 horas de período afebril?	Physician
Sore throat	Do you have a sore throat?	Você tem dor de garganta?	Patient
Tachypnoea	Are you breathing faster or with difficulty?	Você está respirando mais rápido ou com dificuldade?	Physician
Vomit	Have you vomited?	Você vomitou?	Patient

 Rev Inst Med Trop São Paulo. 2026;68:e7

Supplementary Table S2 - Main SARS-CoV-2 lineage groups identifier

Month/Year	SARS-CoV-2 Lineages						
	**B.1.1.28**	**B.1.1.33**	**Others***	**Gamma ****	**Zeta**	**Total**	**Phase**
April/2020	59	19	1			79	Phase 1
May/2020	59	10	4		1	74	
Jun/2020	29	16	1			46	
July/2020	37	6	2			45	
Aug/2020	22	7	3			32	
Sept/2020	13		1			14	
Oct/2020	43	4	5		2	54	Phase 2
Nov/2020	34	5	10		16	65	
Dez/2020	57	7	21		26	111	
Jan/2021	27	1	17	16	43	104	Phase 3
Feb/2021	5	1	6	69	6	87	
Mar/2021	2	1	6	124	9	142	
April/2021	1		3	22		26	
**Total**	**388**	**77**	**80**	**231**	**103**	**879**	

*Other: B.1.1, B.1.1.174, B.1.1.348, B.1.1.135, B.1.1.371, B.1.1.274, B.1.1.397, N9, P7, B1.1.7, B.1.1.10.; **Gamma group: P.1, P.1.10, P.1.14.

Supplementary Table S3 - Relative risk of the occurrence of a given symptom using as reference the prevalence of that symptom for lineages B.1.1.28 and B.1.1.33. Benjamini-Hochberg correction with a 10% false positive rate rejects all hypothesis tests with p<0.0033. An asterisk was added after the p-values to indicate that the hypothesis test was rejected

Symptom	Gamma vs B.1.1.28 and B.1.1.33		Zeta vs B.1.1.28 and B.1.1.33	
	**Relative risk (95%CI)**	**p-value**	**Relative risk (95%CI)**	**p-value**
Ageusia	0.81 (0.70 – 0.93)	2.15E-03 *	0.96 (0.80 – 1.11)	6.49E-01
Altered mental status	0.46 (0.15 – 1.12)	1.20E-01	2.09 (0.95 – 4.17)	8.07E-02
Anorexia	0.98 (0.86 – 1.11)	8.06E-01	0.88 (0.71 – 1.06)	2.25E-01
Anosmia	0.80 (0.69 – 0.91)	9.38E-04 *	0.90 (0.74 – 1.06)	2.14E-01
Coryza/Stuffy nose	0.82 (0.74 – 0.90)	1.62E-05 *	0.96 (0.85 – 1.05)	4.76E-01
Cough	1.07 (1.00 – 1.13)	4.71E-02	1.01 (0.91 – 1.10)	8.81E-01
Dyspnoea	1.81 (1.22 – 2.66)	3.32E-03	1.79 (1.07 – 2.83)	3.75E-02
Fatigue	1.02 (0.95 – 1.09)	5.79E-01	0.98 (0.88 - 1.07)	7.71E-01
Fever	0.99 (0.89 – 1.10)	9.31E-01	0.89 (0.74 – 1.03)	1.35E-01
Headache	0.98 (0.91 – 1.04)	5.72E-01	0.95 (0.85 – 1.04)	2.91E-01
Joint pain	0.99 (0.85 – 1.14)	9.35E-01	1.13 (0.94 – 1.33)	1.91E-01
Myalgia	1.03 (0.95 – 1.11)	4.15E-01	0.99 (0.88 – 1.10)	1.00E+00
Nausea	0.97 (0.77 – 1.22)	7.96E-01	0.99 (0.71 – 1.32)	1.00E+00
Persistent fever	1.85 (1.40 – 2.45)	4.08E-05 *	1.61 (1.08 – 2.35)	2.35E-02
Sore throat	1.08 (0.96 – 1.21)	2.04E-01	1.12 (0.96 – 1.28)	1.72E-01
Tachypnoea	0.64 (0.33 – 1.18)	1.98E-01	0.57 (0.20 – 1.30)	2.04E-01
Vomit	1.05 (0.64 – 1.66)	8.92E-01	1.23 (0.65 – 2.15)	5.86E-01

Supplementary Table S4 - Relative Risks and p-values for the occurrence of altered mental status. In each column, the second lineage displayed is used as reference for calculating relative risks. A Benjamini-Hochberg correction with a 10% false positive rate rejects all hypothesis tests with p<0.050. An asterisk was added after the p-values to indicate that the hypothesis test was rejected

	Zeta vs B.1.1.28	Zeta vs B.1.1.33	Zeta vs Gamma	B.1.1.28 vs B.1.1.33	Gamma vs B.1.1.28	Gamma vs B.1.1.33
Relative Risk (95% CrI)	1.75 (0.80 – 3.51)	11.26 (1.79 – 302.6)	4.55 (1.64 – 14.71)	6.42 (1.16 – 186.29)	0.38 (0.12 – 0.94)	2.33 (0.32 – 73.82)
p-value	0.2375	0.0053*	0.0045*	0.0327*	0.0500*	0.5759

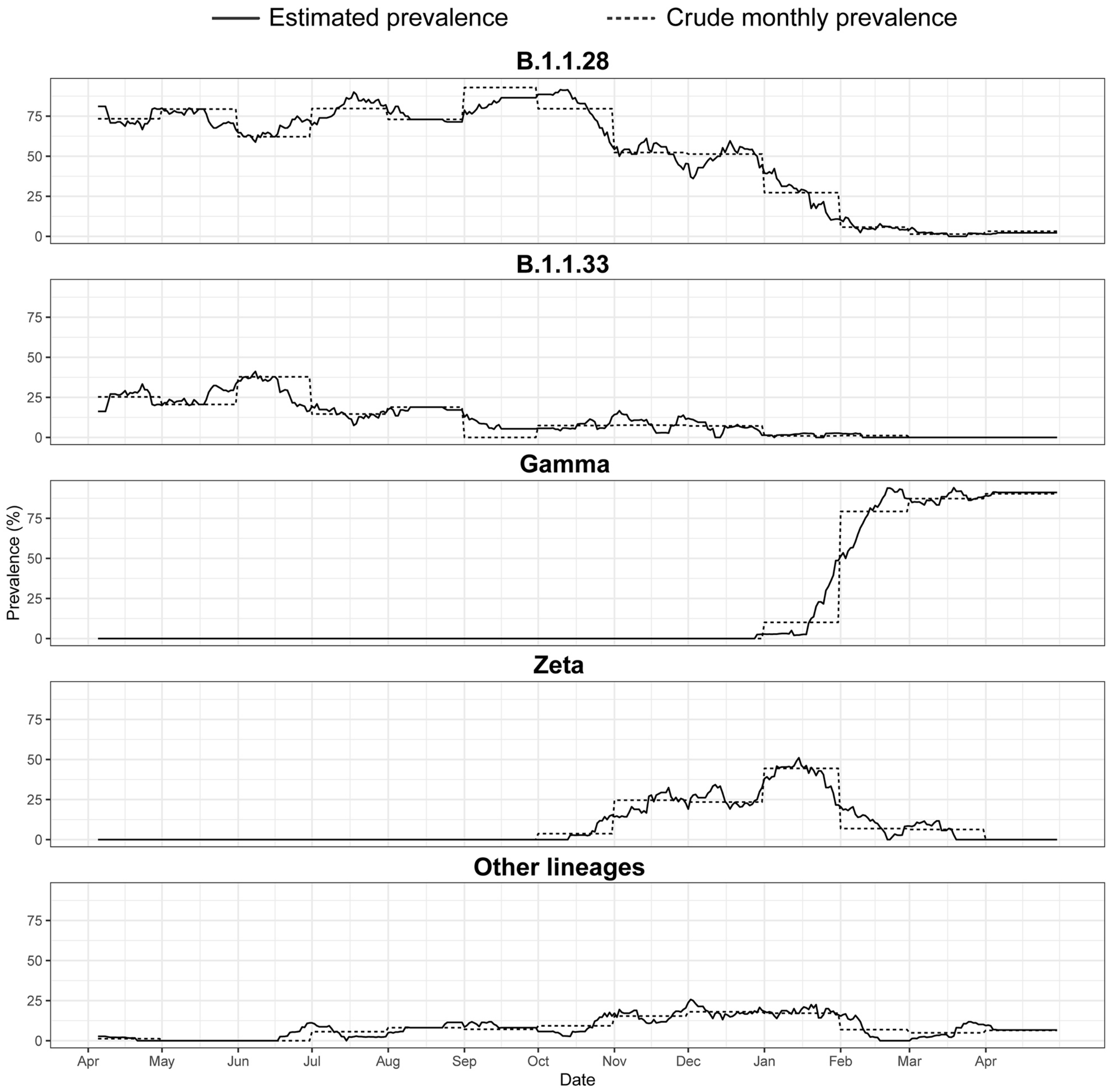

Supplementary Figure S1 - Estimated lineage prevalence compared with the crude monthly lineage prevalence, defined as the ratio between the number of samples of a given sequence in each month and the total number of samples in the same month.

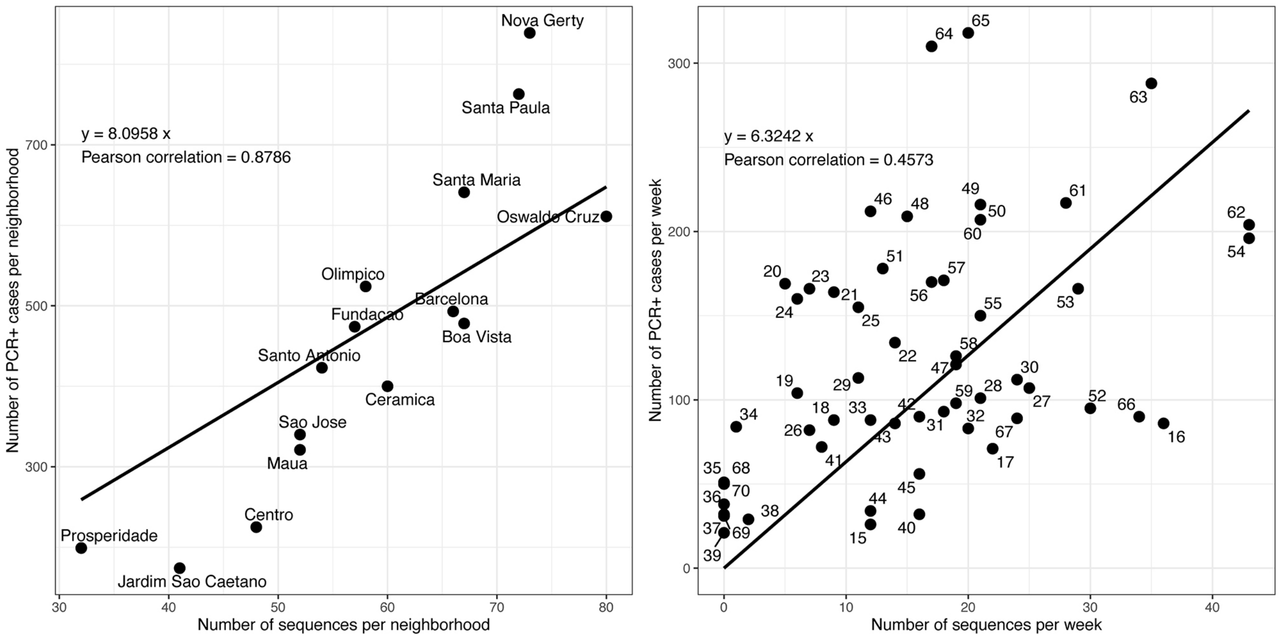

Supplementary Figure S2 - Correlation between number of PCR+ cases and number of sequences aggregated by neighbourhood (A) and month (B). Number of genomes strongly correlates with number of SARS-CoV-2 PCR+ confirmed cases both spatially and temporally.

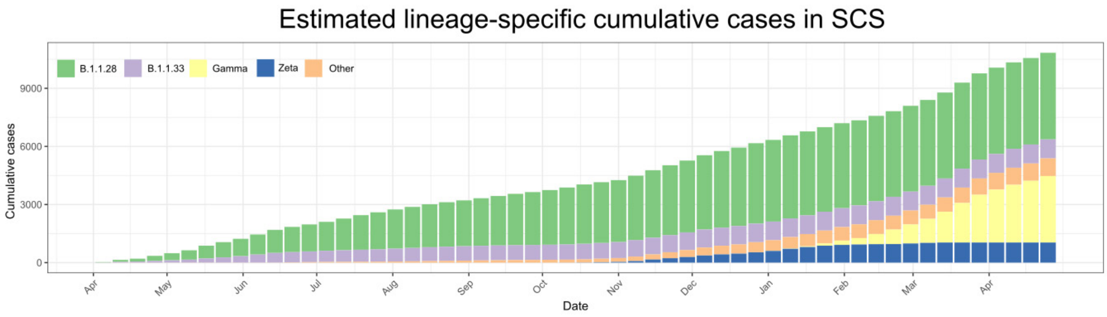

Supplementary Figure S3 - Estimates for cumulative cases caused by each lineage in Sao Caetano do Sul. By April 30, 2021, 41.3% of the cases were caused by B.1.1.28, followed by Gamma (31.7%), Zeta (9.6%), and B.1.1.33 (9.0%).

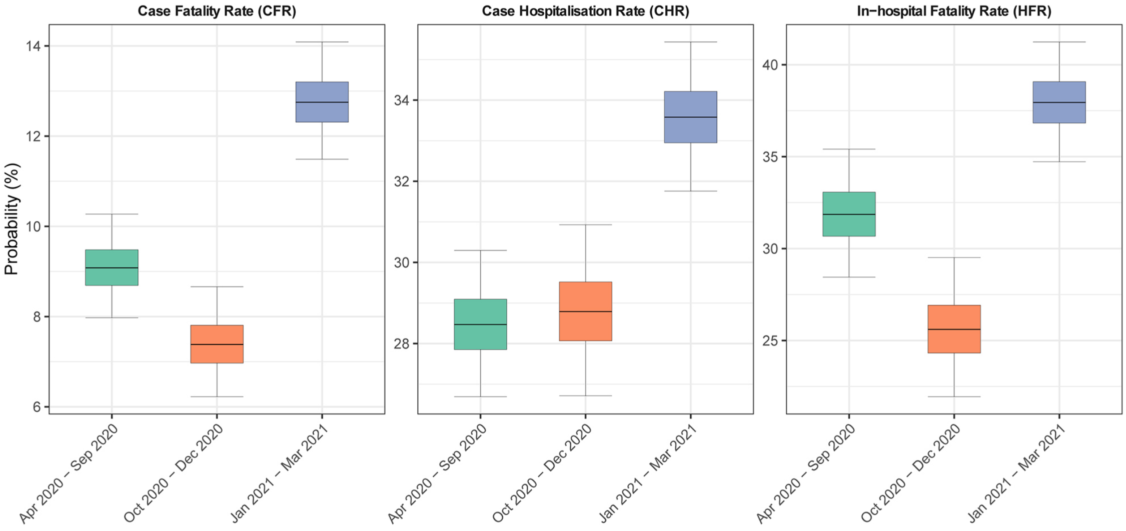

Supplementary Figure S4 - Inferred case fatality rate (CFR), hospitalisation rate (CHR), and in-hospital fatality rate (HFR) disaggregated by study period. All these indicators were higher during the third period when Gamma VOC was prevalent.

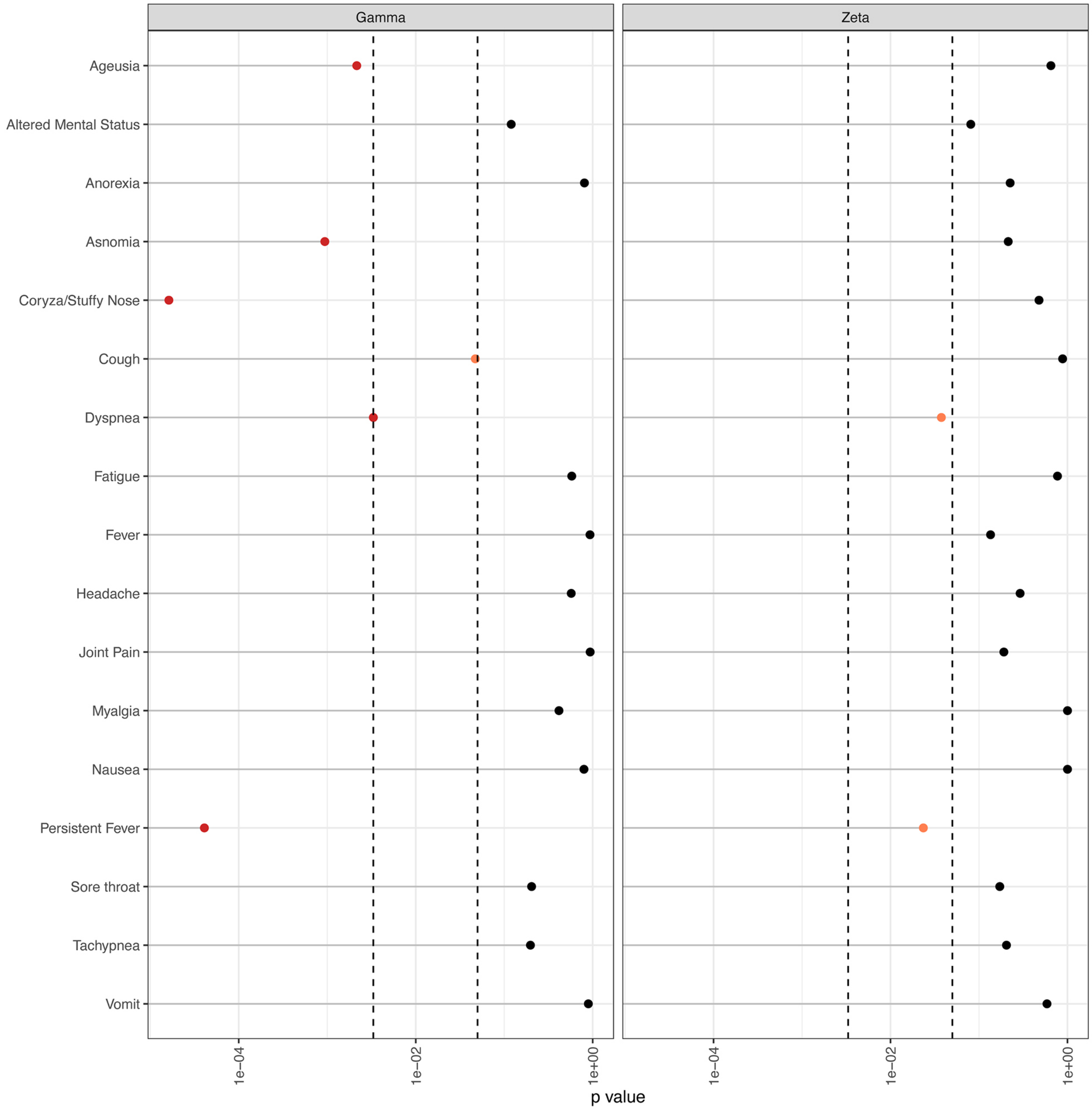

Supplementary Figure S5 - P-values obtained by testing if a patient infected by a given VOC or VOI (Gamma or Zeta) is more likely to develop a given symptom than B.1.1.28 and B.1.1.33. We used Fisher's exact test with a 95% confidence level (points in red or orange). Since 34 hypothesis tests were performed, a Benjamini-Hochberg correction was applied with a 10% false positive rate which rejected all hypothesis tests with $p \leq 0.0033$ (in red).

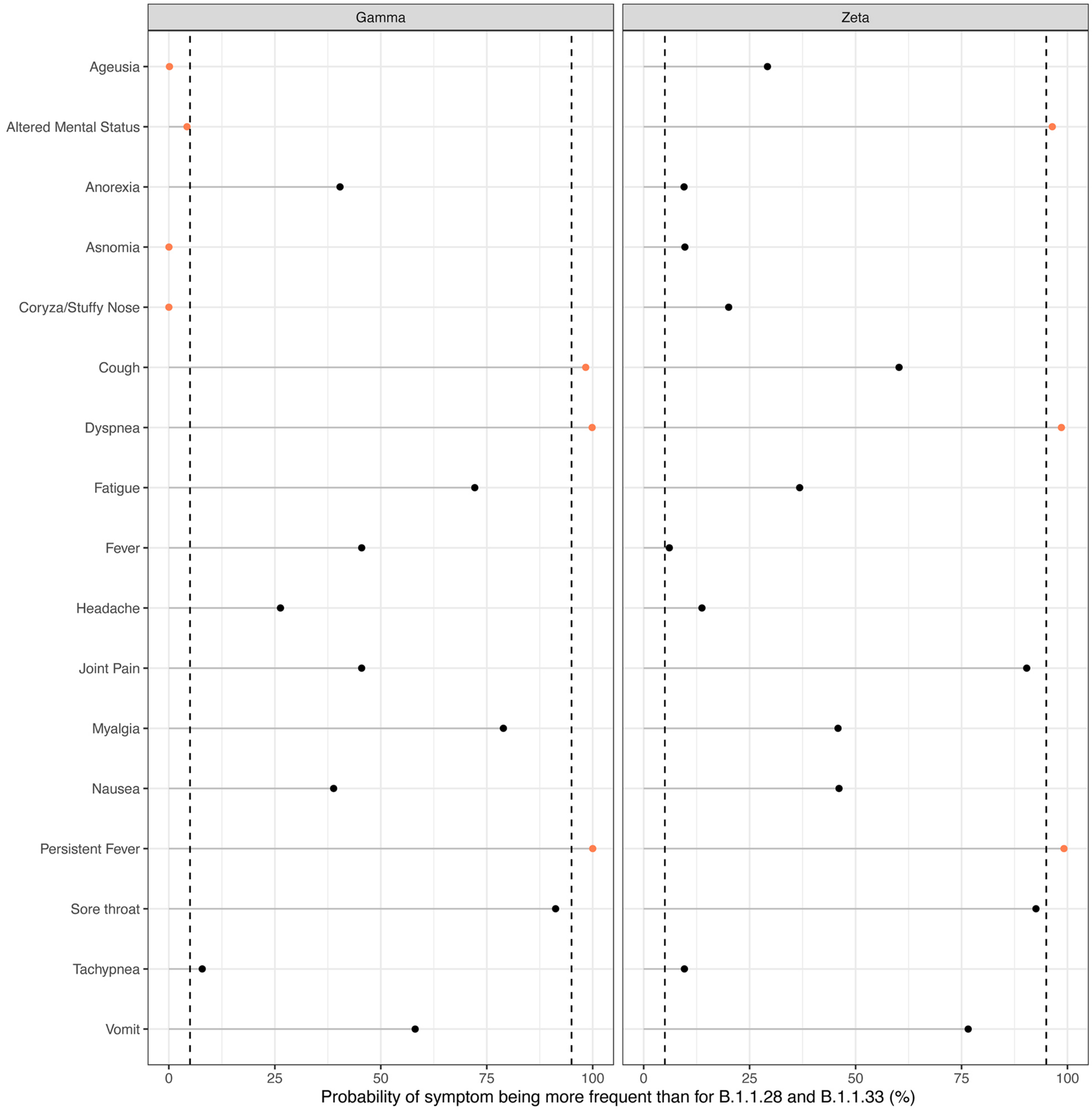

Supplementary Figure S6 - Probability of a given symptom being more frequently caused by Gamma or Zeta compared with B.1.1.28 and B.1.1.33. Dashed lines represent probabilities of 5% and 95%, and red points represent symptoms with probabilities over these thresholds. For example, ageusia, altered mental status, anosmia, and coryza/stuffy nose are more likely to occur in patients infected by B.1.1.28 and B.1.1.33 than by Gamma. On the other hand, patients infected by Gamma are more likely to develop cough, dyspnoea, and persistent fever than patients infected by B.1.1.28 and B.1.1.33. See methods for details on how probabilities were calculated.

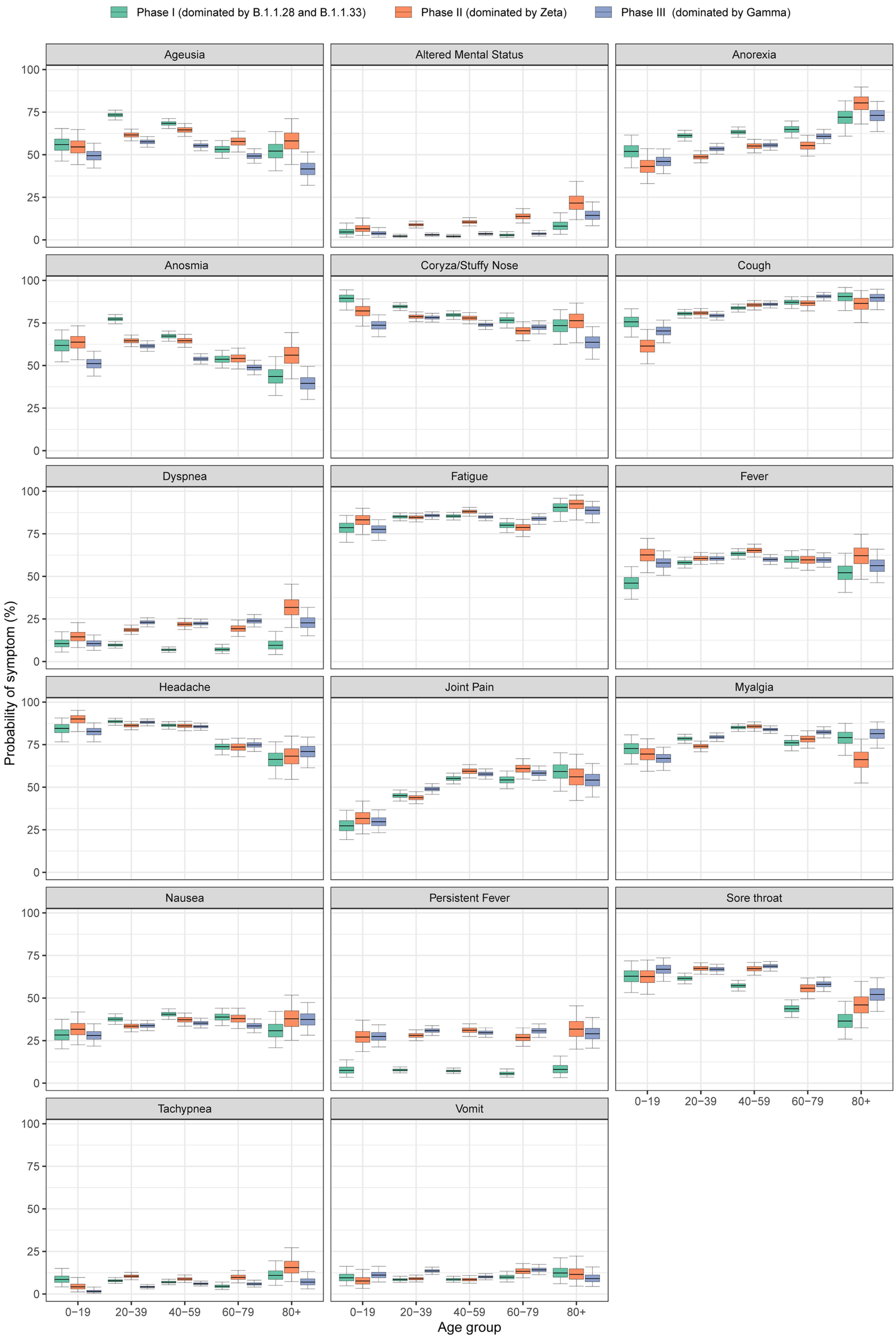

Supplementary Figure S7 - Probability of occurrence of a given symptom by age group and phase of study (I, II, III). Boxes show the median and 50% credible intervals, and whiskers indicate the 95% credible intervals. Warning signs for severity: fever/persistent fever, dyspnoea/difficulty breathing and tachypnoea, altered mental status.
